# Molecular docking analysis of oxazole compounds with the heme-binding protein from *Porphyromonas gingivalis*

**DOI:** 10.6026/97320630019105

**Published:** 2023-01-31

**Authors:** Pranaw Manikandan, Vishnu Priya Veeraraghavan, Surya Sekaran, Gayathri Rengasamy, Rajalakshmanan Eswaramoorthy

**Affiliations:** 1 Department of Biochemistry, Saveetha Dental College and Hospitals, Saveetha Institute of Medical and Technical Sciences, Saveetha University, Chennai-600077, India; 2 Department of Biomaterials (Green lab), Saveetha Dental College and Hospital, Saveetha Institute of Medical and Technical Science (SIMATS), Saveetha University, Chennai-600077, India

**Keywords:** Auxotrophic bacteria, Computational analysis, In Silico,, Heme binding protein, Periodontitis, *P. gingivalis*

## Abstract

*Porphyromonas gingivalis*, a peripathogen, has several methods to impede or modify the protective mechanisms of the teeth. Targeting the inhibition of the heme protein will prevent the organism from multiplying and inhibit the virulence mechanism. The literature derived oxazole compounds (1-5) were docked against the protein's active site, and the results show that the selected oxazole derivatives exhibit better interaction compared to clinically proven drugs.

## Background:

A major pathogen in chronic periodontitis is *P. gingivalis*. In other words, it modifies the local microbial community's size and makeup to encourage periodontitis and cause an inflammatory environment. Additionally, it has the capacity to enter the circulation and colonize extraoral regions [1]. Periodontitis is mostly brought on by the pathogen *Porphyromonas gingivalis* (*P. gingivalis*) is an important pathogenic bacterium that plays a role in periodontitis' local inflammatory response in P.gingivalis [2]. Foreign protein and polypeptides that give *P. gingivalis* nourishment and support its growth can be broken down by gingipains, a gram-negative, obligatory anaerobic bacillus is *P. gingivalis* [3]. It has the ability to produce a number of virulence factors, such as lipopolysaccharides, extracellular polysaccharides, trichoderma, gingipains, tetratricopeptide repeat (TPR) sequence protein, and gingipains [4]. Inflammation brought on by gingipains and LPS co-activation may disrupt the immune defense mechanisms of periodontal tissues, resulting in the death of periodontal tissues and alveolar bone resorption [5]. One of the keystone pathogens among the periodontal bacteria that cause illness is *P. gingivalis* [6]. The various ways that *P. gingivalis* inhibits innate immunity could change the composition of the biofilm and cause a detrimental change in the typically homeostatic host-microbial balance [7]. Instead of directly causing damage to periodontal tissues, *P. gingivalis* may encourage neighboring bacteria to induce damaging inflammation. As a result, *P. gingivalis* is thought to perform a role as a keystone pathogen that disturbs host-commensal balance [8]. Being a heme auxotroph, *P. gingivalis* must obtain heme as a source of iron (Fe) and protoporphyrin IX (PPIX). TonB-dependent outer-membrane receptor (HmuR), which transports heme through the outer membrane, receives heme from host hemoproteins or heme-binding proteins generated by coexisting bacteria through the action of HmuY [9]. The proteins encoded by multiple distinct species of the Bacteroidetes phylum, which is divided into three classes: Bacteroidetes, Flavobacteria, and Sphingobacteria, are comparable to HmuY [10]. Several human diseases, particularly those that infect the oral cavity, contain bacteria that encode putative HmuY homologues. An essential method by which *P. gingivalis* and other pathogenic bacteria acquire these substances for their survival and capacity to cause an infection is by the uptake of heme as iron and protoporphyrin IX [11]. HmuR and HmuY proteins make up the heme acquisition systems in *P. gingivalis*.A heme-binding lipoprotein called HmuY is connected to the bacterial cell's outer membrane. As part of the heme acquisition process, oxyhemoglobin is first converted to methemoglobin. Oxyhemoglobin first undergoes a conversion to methemoglobin as a part of the heme acquisition process. The HmuY family of proteins binds heme using a variety of amino acid residues. *P. gingivalis*. HmuY has histidine residues (H134 and H166; amino acid residue numbers are based on full-length, unprocessed proteins) that coordinate the heme iron [12]. With the help of *P. gingivalis* cysteine protease and lysine-specific gingipain K (Kgp) activity, bacteria are able to totally proteolyze the more sensitive methemoglobin to release free heme. The oral microbiome's commensal organisms may also help periodontitis-related bacteria, particularly *P. gingivalis*, which is better adapted to both anaerobic and aerobic circumstances [13]. Modern medicinal chemistry techniques, such as molecular modeling, have been used more frequently by the research-based pharmaceutical industry as effective tools for the study of structure-activity relationships (SAR). Through the use of these approaches, pharmacokinetic properties (ADMET: absorption, distribution, metabolism, excretion, and toxicity) as well as pharmacodynamics data (e.g Potency, affinity, efficacy, selectivity) have been examined [14]. An increasing need for reliable and advanced computational tools has been created by efforts in storing, organizing, and exploring this information. According to this viewpoint, the precise fusion of in-silico and experimental methodologies has given rise to the most recent knowledge of the complex facets of intermolecular recognition. Structure-based drug design (SBDD) techniques, or the utilization of three-dimensional structural data acquired from biological targets, constitute a significant part of contemporary medicinal chemistry within this framework [15]. The most widely used SBDD techniques include molecular docking, structure-based virtual screening (SBVS), and molecular dynamics (MD), which have a variety of uses in the analysis of molecular recognition events like binding energetics, molecular interactions, and induced conformational changes. Bioactive small-molecule libraries are a distinctive strategy in medication design. The space occupied by ligands known to interact with a particular target is represented by the distinctive chemical diversity contained in these libraries. Methods for ligand-based drug design (LBDD) employ this kind of data [16]. The ANCHOR algorithm locates potential binding pockets with druggable properties by looking for amino acid side chains buried deeply at protein-protein interfaces (anchor residues). These anchor sites, unlike hotspots, carry explicit concave/convex surfaces, which is a useful property for ligand binding. They have the capacity to produce a strong attraction between the receptor and the ligand. As they may lead to treatment failures and the withdrawal of medications from the market, adverse drug reactions (ADRs) are one of the key concerns in pharmaceutical research and drug development. ADRs may result from factors relating to the medicine or to the unique patient characteristics. These reactions can be brought on by a number of factors in relation to medications, including dosage, drug formulation, method of administration, drug-drug interactions (DDI), drug-food interactions, drug metabolism, and allergic or hypersensitive reactions that have an impact on the immune system [17]. Therefore, the aim of this study is to identify potential drug candidates to inhibit HmuY (Heme binding protein) of *Porphyromonas gingivalis*.

##  Materials and methods:

## Selection of drug inhibitors (HmuY Protein inhibitor):

Five Oxazole derivatives, compounds (1-5) that are not yet clinically used are derived from previous literature.

## Preparation of Ligands:

**Figure 1:**
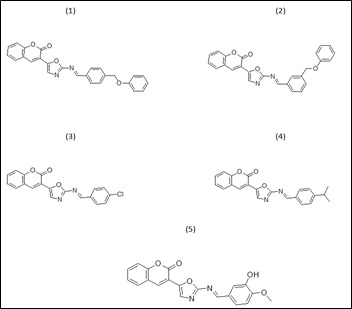
2D structure of the oxazole compounds (1-5)

The 2D mol structures of the selected compounds were prepared using ChemOffice Suite 16.0 (Figure 1). During the optimization method, all parameters were selected in order to achieve a stable structure with the least amount of energy. Each molecule's 3D coordinates (PDB) were determined using optimized structure.

## Preparation of Macromolecule:

**Figure 2:**
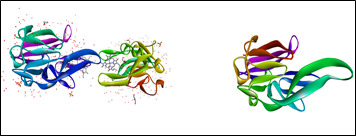
3D structure of the heme binding protein and prepared protein of *P. gingivalis*

The 3D structure (Figure 2) of the heme binding of P.gingivalis was retrieved from the protein data bank (PDB Id: 3H8T). Water molecules, other hetero atoms, co-crystallized ligands were removed, and the protein was prepared by adding polar hydrogens and kollman charges in accordance with the standard protocol employing the software Biovia Discovery Studio and Mgl tools (1.5.7).

## Molecular docking:

**Figure 3:**
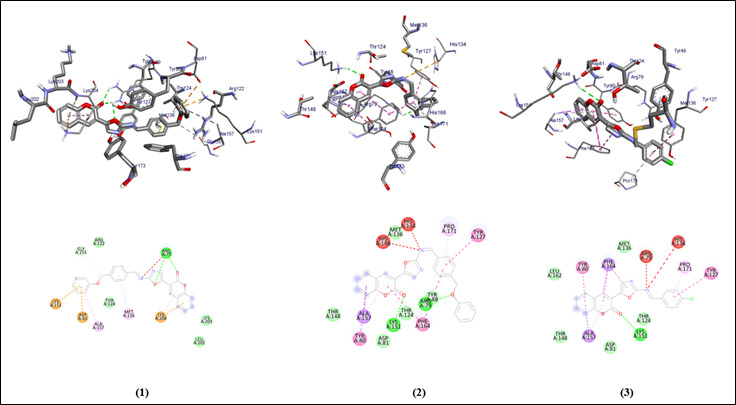
Molecular docking analysis of compounds (1-3) against the target Heme binding protein of *Porphyromonas gingivalis*

**Figure 4:**
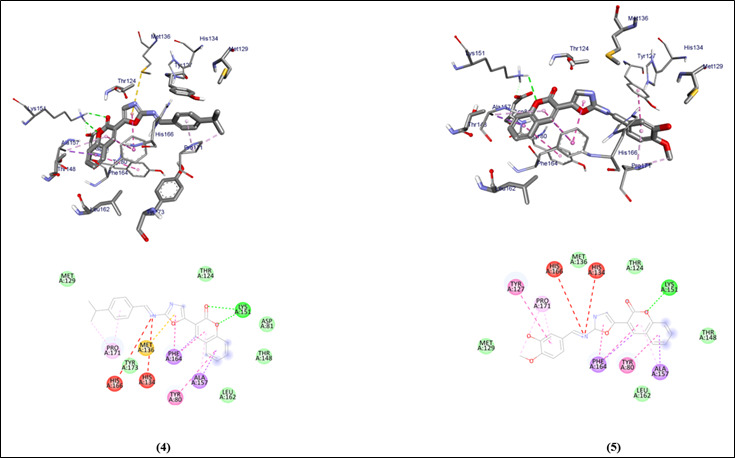
Molecular docking analysis of compounds (4 & 5) against the target Heme binding protein of *Porphyromonas gingivalis*.

The graphical user interface Auto Dock vina was used for Ligand-Protein docking interactions ( Figure 3 - Figure4). Auto Dock Tools (ADT), a free visual user interface (GUI) for the AutoDock Vina software, was used for the molecular docking research. The oxazole compounds (1-5) were docked against the protein's active site, AutoDockVina was employed with a grid point center spacing of -9.515, 26.270, and 22.0381 along the x, y and z axis respectively. The dimensions (Angstrom) of the grid box are 42.688, 47.783, and 39.555 that point in the x, y, and z directions respectively. For each ligand, nine alternative conformations were created and ranked based on their binding energies using the AutoDockVina scoring functions. The post-docking evaluations were conducted using PyMOL and AutoDock Tools.

## Evaluation of ADMET:

The SwissADME and PROTOX online servers were used for estimating the absorption, distribution, metabolism and excretion so that we can effectively determine their side effects and avoid any harm. SwissADME shows whether the selected ligands are inhibitors of important cytochromes that play an important role in our metabolism processes and enzyme activities (CYP1A2, CYP2C19, CYP2C9, CYP2D6, and CYP3A6), whether the ligands have good gastro intestinal absorption, are they permeable to the blood brain barrier. PROTOX II is standard online software to check the toxicity of our drug with our compound's chemical formula and structure. It calculates the toxicity of our compound. The ligands are checked for hepatotoxicity, carcinogenicity, immunotoxicity, mutagenicity, and according to all the tests the lethal dosage at which the drug can be given is calculated in mg/Kg.

## Statistical analysis:

ANOVA (p<0.05), the docking results are compared with the clinically used positive control group (Amoxicillin, Moxifloxacin, sulfanilamide and sulfamethoxazole. The ligands (oxazole derivatives) are compared with already existing drugs to analyze its efficiency and potency to inhibit the HmuY protein.

## Results:

## Molecular docking interaction of oxazole compounds against Heme binding protein of *P. gingivalis*:

Ligand-Protein interaction of all the five oxazole compounds (1-5) with the heme binding protein is compared with the already existing standard drugs for *P. gingivalis*. Their potential to inhibit them is analyzed. The docking affinity scores of compounds (1-5) show better affinity values (-10, -11.3, -9.6, -10, -9.4) when compared to already existing drugs Amoxicillin, Moxifloxacin, Sulfanilamide and Sulfamethoxazole (-8.6, -8.6, -6, -8.1). The hydrogen bond interactions of the compound 2 are more and stronger when compared to all the existing standard drugs. The compounds (1-5) have stable H bonding, weak hydrophobic and Van dar Waals interaction within the binding pocket of the proteins (Table 1).

## ADME and *Lipinski's rule of five*:

Compounds 1-5, all obey Lipinski's rule of five, same as the clinically proven drugs Amoxicillin, and Moxifloxacin (Table 3). All these compounds have molecular weight < 500, iLogP value < 5, HBD value <5, Lipinski violations are zero. The compounds show log Kp values between -5.26 to -6.2 cm/s. All the compounds show high gastro intestinal absorption so it doesn’t need a carrier molecule (Table 2). The bioavailability score of all these compounds are 0.55 same as already existing drugs. Compounds 1-5 do not inhibit cytochromes CYP2D6, CYP3A6 and their Gastro-intestinal absorption is very high, same as Amoxicillin. Except compound 3 all other drugs are not permeable to Blood brain barrier same as Amoxicillin.

##  Toxicity profiling:

The lethal dosage values of compounds 3, 4 (2500mg/Kg) are in the same range as the drugs Moxifloxacin and Sulfamethoxazole (200, 2300 mg/Kg). The compounds 1-4 show no immunotoxicity, same as the already existing standard drugs (Table 4). All the compounds show carcinogenicity and none of the compounds show cytotoxicity. Compounds 1-4 show mutagenicity the same as Amoxicillin, Sulfanilamide and Sulfamethoxazole.

## Discussion:

The selected compounds (oxazole derivatives) 1(-10) to 5(-9.4) show better interaction compared to the clinically proven drugs (-6Kcal, -8.6Kcal) [18]. Compounds 1, 3, 4, 5 follows Lipinski's rule of five similar to sulfonamide and moxifloxacin. Compound 2 does not follow Lipinski's Rule of 5 similar to sulfonamide and moxifloxacin and amoxicillin. All the compounds have good skin permeability (logKp -5.11 to -5.42) and all five have high gastro intestinal absorption [19]. The toxicity profile of the drugs is a little high. They all range between class 4 to class 6. The reported compounds have stable H-bonding, weak hydrophobic and Van dar Waals interactions within the binding pocket of the proteins. They all have high efficiency to inhibit the HmuY protein but the toxicity of the drugs is also a little high. Further research on reducing the toxicity of the drugs and giving them in correct dosages will lead to finding an efficient drug to cure diseases caused by P .gingivalis [20].

## Conclusion:

The selected ligands (1-5) show optimal interactions with the heme binding protein within the binding sites. All obey Lipinski's rule of five and have excellent interaction scores when compared to already existing drugs for *Porphyromonas gingivalis* (amoxicillin, moxifloxacin, etc.) for further consideration in drug discovery. All the selected ligands show good docking affinity, hydrogen bond interactions and hydrophilic interactions. Among the selected ligands compound 2 has the highest affinity score and has the potential to act as a potential inhibitor for heme binding protein of *P. gingivalis*.

## Figures and Tables

**Table 1:** Molecular docking scores and residual amino acid interactions of Oxazole compounds (1-5) against heme-binding protein Hmu Y of *Porphyromonas gingivalis* (PDB ID: 3H8T).

Ligands	Docking scores/Affinity (kcal/mol)	H-bond	Amino Acid Residual interactions	
			Hydrophobic/Pi-Cation	Van dar Waals
1	-10	Arg-79	Lys-204, Asp-81, Lys-151, Ala-157, Met-136	Arg-122, Thr-124, Leu-202, Lys-203, Gly-155
2	-11.3	Thr-148, Thr-124, Asp-81, Tyr-48, Met-136	His-166, His-134, Ala-157, Tyr-127, Tyr-80, Phe-164, Pro-171	Lys-151, Arg-79
3	-9.6	Lys-151	Tyr-80, Phe-164, Ala-157, Arg-79, His-134, Pro-171, Tyr-127	Leu-162, Thr-148, Asp-81, Thr-124, Met-136
4	-10	Lys-151	Pro-171, His-166, His-134, Met-136, Phe-164, Ala-157, Tyr-80	Met-129, Thr-124, Asp-81, Thr-148, Leu-162, Tyr-173
5	-9.4	Lys-151	Tyr-127, Pro-171, His-166, His-134, Ala-157, Tyr-80, Phe-164	Met-136, Met-129, Thr-124, Thr-148, Leu-162
Amoxicillin	-8.6	Tyr-173, Arg-79, Tyr-80, Asp-81	Pro-171	His-78, Ala-157, Lys-151, Thr-124, Tyr-48, Tyr-127, His-166, His-134, Met-129
Moxifloxacin	-8.6	Gln-154, Arg-122	His-134, Phe-164, Met-136, Pro171, Lys-204	Gly-155, Thr-124, Tyr-48, Tyr-80, His-166, Phe-156
Sulfanilamide	-6	Tyr-48, Thr-124, Asp-81	Arg-79	His-78, Arg-122, Lys-151, Tyr-80
Sulfamethoxazole	-8.1	Gly-155, Lys-151, Thr-124, Tyr-48,	Leu-162, Phe-164, Ala-157, Tyr-80	Asp-81, Arg-122, Arg-79, Tyr-127

**Table 2:** SwissADME values of selected oxazole compounds (1-5)

Compound	log Kp (cm/s)	GI absorption	BBB permeant	Pgp substrate	CYP1A2 inhibitor	CYP2C19 inhibitor	CYP2C9 inhibitor	CYP2D6 inhibitor	CYP3A4 inhibitor
1	-5.26	High	No	No	No	Yes	Yes	No	No
2	-5.26	High	No	No	No	Yes	Yes	No	No
3	-5.42	High	Yes	No	Yes	Yes	Yes	No	No
4	-5.11	High	No	No	Yes	Yes	Yes	No	No
5	-6.2	High	No	No	Yes	No	Yes	No	No
Amoxicillin	-9.94	Low	No	No	No	No	No	No	No
Moxifloxacin	-8.32	High	No	Yes	No	No	No	Yes	No
Sulfanilamide	-7.79	High	No	No	No	No	No	No	No
Sulfamethoxazole	-7.21	High	No	No	No	No	No	No	No

**Table 3:** Lipinski and Veber rules of selected oxazole compounds (1-5)

Compound	MW	iLogP	HBD (nOHNH)	HBA (nON)	nrotb	MR	TPSA	Lipinski #violations	Bio availability score
Lipinski*	≤500	≤5	≤5	≤10	≤10	-	-		
Veber**	-	-	-	-	-	-	≤ 140		
1	422.43	4.17	0	6	6	122.66	77.83	0	0.55
2	422.43	4.21	0	6	6	122.66	77.83	0	0.55
3	350.76	3.26	0	5	3	96.69	68.6	0	0.55
4	358.39	3.61	0	5	4	106.26	68.6	0	0.55
5	362.34	3.02	1	7	4	100.19	98.06	0	0.55
Amoxicillin	365.4	1.46	4	6	5	94.59	158.26	0	0.55
Moxifloxacin	401.43	2.78	2	6	4	114.05	83.8	0	0.55
Sulfanilamide	172.2	0.61	2	3	1	41.84	94.56	0	0.55
Sulfamethoxazole	253.28	1.03	2	4	3	62.99	106.6	0	0.55

**Table 4:** Toxicity profile of selected oxazole compounds (1-5)

			Toxicity				
Compound	^a^LD_50_ (mg/kg)	Class	HEPATOTOXICITY	CARCINOGENICITY	IMMUNOTOXICITY	MUTAGENICITY	CYTOTOXICITY
1	1127	4	Active	Active	Inactive	Inactive	Inactive
2	1127	4	Active	Active	Inactive	Inactive	Inactive
3	2500	5	Active	Active	Inactive	Inactive	Inactive
4	2500	5	Active	Active	Inactive	Inactive	Inactive
5	500	4	Active	Active	Active	Active	Inactive
Amoxicillin	15000	6	Inactive	Inactive	Inactive	Inactive	Inactive
Moxifloxacin	2000	4	Inactive	Inactive	Inactive	Active	Inactive
Sulfanilamide	3000	5	Inactive	Active	Inactive	Inactive	Inactive
Sulfamethoxazole	2300	5	Active	Active	Inactive	Inactive	Inactive
^a^LD_50_: lethal dose parameter
